# Design and Testing of Root-Specific Synthetic Promoters by Machine Learning

**DOI:** 10.3390/ijms27062540

**Published:** 2026-03-10

**Authors:** Chunhao Lu, Yuepeng Song, Deqiang Zhang

**Affiliations:** 1 State Key Laboratory of Tree Genetics and Breeding, College of Biological Sciences and Technology, Beijing Forestry University, Beijing 100083, China; chunhaolu@bjfu.edu.cn (C.L.); yuepengsong@bjfu.edu.cn (Y.S.); 2 National Engineering Research Center of Tree Breeding and Ecological Restoration, College of Biological Sciences and Technology, Beijing Forestry University, Beijing 100083, China; 3 Key Laboratory of Genetics and Breeding in Forest Trees and Ornamental Plants, Ministry of Education, College of Biological Sciences and Technology, Beijing Forestry University, Beijing 100083, China; 4 College of Horticulture and Landscape Architecture, Beijing Vocational College of Agriculture, Beijing 102442, China

**Keywords:** cis-regulatory module, machine learning, *Populus*, synthetic promoter

## Abstract

Synthetic promoters are crucial for precise gene expression in transgenic plants, but their rational design is hindered by the difficulty in identifying functional cis-regulatory elements (CREs). In this study, we aimed to develop a systematic approach for discovering tissue-specific cis-regulatory modules (CRMs) and generating functional synthetic promoters in poplar. We performed extensive transcriptomic analysis across various poplar tissues to obtain categorical labels and detected motifs in all gene promoters using known transcription factor binding site (TFBS) position weight matrices. Informative, tissue-specific TFBSs were predicted using a random forest model. Applying this to a root-specific gene, *PopRTS1*, we identified putative root-specific CRMs. These CRMs were then used to construct synthetic promoters, which were experimentally validated through rapid infiltration and GUS staining assays across different tissues. We successfully identified a root-specific synthetic promoter, PRTS1. Our findings demonstrate that machine learning can effectively decipher regulatory codes from omics data to predict functional CRMs. This work provides a feasible and systematic method for screening and designing tissue-specific synthetic promoters, offering significant potential for advancing targeted gene expression systems in plant biotechnology.

## 1. Introduction

Synthetic biology (synbio) holds the potential to revolutionize genetic engineering for the purpose of sustainability. Among the cutting-edge synbio techniques, genome editing, gene circuit design, synthetic promoter development, gene stacking technologies, and environmental sensor design stand out as particularly promising. In the realm of plant biotechnology, synthetic promoters have emerged as one of the most advanced synbio tools, offering precise solutions for spatiotemporal expression requirements [1]. In order to fine-tune transgene expression, synthetic promoters are typically constructed by assembling a variety of cis-regulatory elements (CREs) in innovative combinations [2]. The primary strategy for designing synthetic promoters involves merging multiple cis-elements or random sequences with a core promoter, followed by screening the resulting synthetic promoters for their suitability in experimental applications [3,4,5]. This process facilitates the development of custom-tailored synthetic promoters, providing a level of precision previously unattainable in various applications.

To date, most synthetic promoters have been constructed by positioning synthetic sequences upstream of a core promoter, such as the widely used 46-bp CaMv 35S promoter (35Smini). A minimal synthetic promoter was recently developed for constitutive response by arranging an eclectic array of common CREs from pathogen promoters in various positions relative to the TATA box region [6]. Additionally, computational models have been employed to optimize native promoter elements within plant core promoters, resulting in enhanced promoter strength [7]. Despite these advancements, pinpointing functional CREs and cis-regulatory modules (CRMs) upstream of a core promoter remains a significant challenge in synthetic promoter design [8]. To address this challenge, high-throughput gene expression profiles and bioinformatic tools have been utilized to aid in the design and testing of synthetic promoters [9]. However, as more and more high-quality sequencing data become available, the time-consuming and error-prone procedure of identifying motifs, designing synthetic promoters, and testing their efficacy persists as a hindrance to achieving greater promoter precision [10].

Recent studies have shown that machine learning (ML) methods hold the potential to serve as a valuable alternative to traditional inferential statistics for deducing the cis-regulatory code from whole-genome annotation data [11,12,13,14,15]. In the context of human promoters, ML has been instrumental in identifying downstream core promoter regions (DPR), DNA- and RNA-binding sequences, and other pertinent features [16,17,18,19]. Comprehensive studies of transcription factor (TF)-binding profiles have also predicted or inferred the cis-regulatory code underlying transcriptional gene regulation in plant research [20,21,22,23]. However, much of the ML work in plants has been directed towards inducible CREs, with only a limited number of studies investigating tissue-specific CREs or CRMs [24]. Recent advances in spatially resolved and single-cell omics have significantly contributed to the discovery and characterization of cell-type-specific mechanisms and spatiotemporal gene regulations in diverse plant tissues, facilitating the expression of cell- and tissue-specific genes [1]. These advancements present promising opportunities for developing more refined and sophisticated ML models capable of deciphering the complexity of cis-regulatory networks in plants.

*Populus* is an economically significant genus and one of the most widely studied tree models. It has been commonly used as a feedstock for manufacturing paper, cellulose, timber, fiber and has potential as a next-generation biofuel [25,26]. The sequencing of the *Populus* genome [27] has enabled routine genetic engineering of the species [9,28]. In this study, we conducted a comprehensive analysis of transcriptomic data from multiple *Populus* tissues with the goal of obtaining categorical labels and identifying potential TF binding sites (TFBSs) in the promoters of all genes in *Populus trichocarpa* and *Populus tomentosa*. We used the TFBSs and promoters of all labeled genes as input data for a random forest model, which allowed us to predict informative TFBSs with tissue-specificity. Using these informative TFBSs, we scanned the promoter of *Pop_A01G005994* (*PopRTS1*), a root-specific gene, and identified putative root-specific CRMs. Leveraging these CRMs, we synthesized a root-specific synthetic promoter, PRTS1, which was later validated in multiple *Populus* tissues using rapid infiltration techniques. The root specificity of this synthetic promoter was verified using β-glucuronidase (GUS) assay. Our study established a reliable and systematic process for identifying tissue-specific CRMs and a feasible method for screening and generating functional synthetic promoters in *Populus*. The findings of this study have significant implications for advancing synthetic biology approaches for forestry improvement and optimizing plant-based industrial processes.

## 2. Results

### 2.1. Transcriptome Profiling of Diverse Tissues of Populus

**Figure 1:**
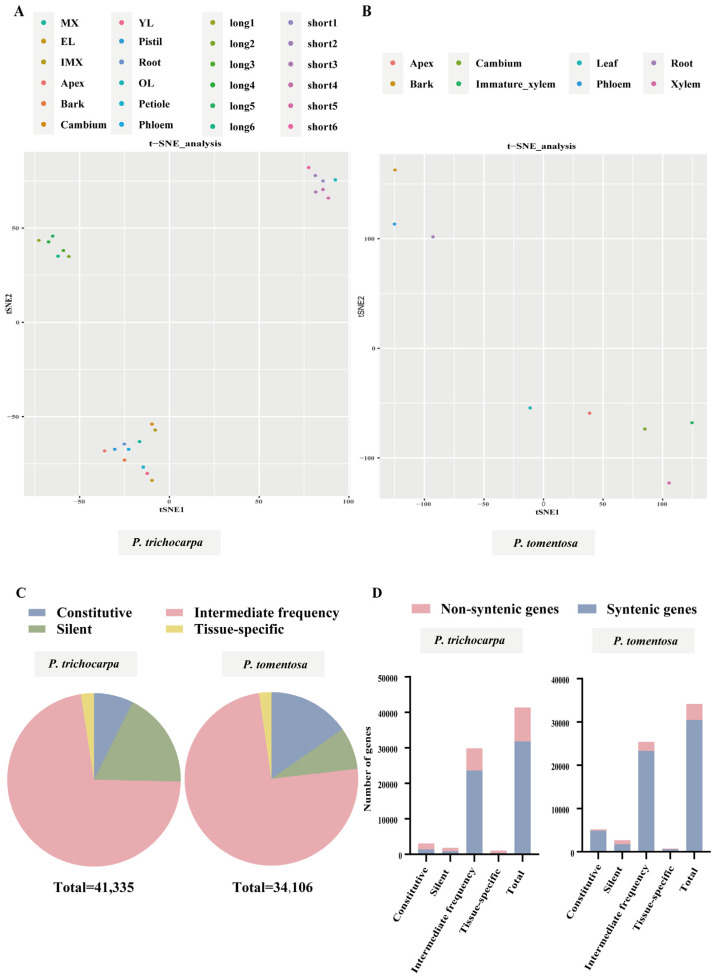
Summary of data collected for this study. (**A**,**B**) t-SNE visualization of all samples in *P. trichocarpa* and *P. tomentosa*. FPKM values of 34,106 genes and 41,335 were used in this analysis. Principal component analysis followed by nearest-neighbor graph-based clustering (t-SNE) was performed to display all samples in 2D space. The color indicates tissue. MX: mature xylem, EL: expanded leaf, IMX: immature xylem, YL: young leaf, OL: old leaf, long 1–6: long branches, short 1–6: short branches. (**C**) Pie chart representing the numbers of genes that are classified into 4 types in two poplars. Different colors represent the proportion out of all genes where each gene is expressed: not expressed in any tissue (Silent, green), expressed with τ = 1.0 (Tissue-specific, yellow), expressed with τ < 0.85 and CV < 0.30 (Constitutive, blue), and the rest is “Intermediate frequency” (pink). (**D**) The proportion of genes in each expression category (defined in (**C**)) that are non-syntenic (relative to other poplars including 84k, *P. tomentosa* and *P. trichocarpa*) were compared with the background gene set (all genes). Blue means syntenic genes.

A total of 24 and eight distinct tissue samples were analyzed for *P. trichocarpa* and *P. tomentosa*, respectively, using RNA-seq profiling, which enabled the categorization of genes with varying expression levels. In *P. trichocarpa*, t-distributed stochastic neighbor embedding (t-SNE) clustering analysis siloed the samples into three clusters based on tissue types. The clustering analysis showed a distinction between long and short branches grouped with old leaves, as well as another cluster made up of residual tissues (Figure 1A). Similar clustering analyses of *P. tomentosa* showed the proximity of the root and phloem (Figure 1B).

*P. trichocarpa* and *P. tomentosa* genomes have 41,335 and 34,106 annotated genes, respectively. According to τ and Coefficient of Variance (CV) values (Figure S1), these genes can be classified into four categories: silent, constitutive expression, tissue-specific expression, and intermediate frequency expression. In *P. trichocarpa*, 18.0% annotated genes were not expressed in any of the tested tissues, and 7.4% showed constitutive expression (τ < 0.85, CV < 0.30). Among all tissue types, 2.4% genes exhibited tissue-specific expression (TSGs, τ = 1), while the remaining were denoted as having an intermediate frequency of expression. In contrast, *P. tomentosa* had only 5.7% of silent genes and 16.0% constitutively expressed genes. According to Zhou et al. [29], it is possible that silent genes may be pseudogenes or genes not expressed in the tissues or environments sampled in this study (Figure 1C).

The proportion of genes in each set that are non-syntenic relative to other poplars was determined for the genes that are tissue specific, constitutive, or silent. *P. tomentosa* had more non-syntenic silent genes (35.8%) than any other poplar species (Figure 1D). On the other hand, tissue-specific and constitutively expressed genes are scarce when compared to the proportion of non-syntenic genes across the entire genome. Although non-syntenic genes made up the majority of silent genes (84.2%), 70.7% of the tissue-specific genes were non-syntenic in *P. trichocarpa* (Figure 1D).

It is plausible that the non-syntenic genes participate in distinct regulatory networks in which TFs play a vital role in regulating gene expression. With this in mind, we analyzed the diversity of TFs across various tissues (Figure S2). Out of a total of 1005 and 788 tissue-specific genes in *P. trichocarpa* and *P. tomentosa*, respectively, only 58 and 70 are TFs, the majority of which were found to be root-specific (Figure S2). Yet, there is scant evidence of a regulatory network connecting root-specific TFs, necessitating additional efforts to tap into the reservoir of TFs implicated in tissue-specific regulation.

### 2.2. Weighted Gene Correlation Network Analysis

To uncover modules of co-expressed genes associated with specific tissues, we utilized the weighted gene co-expression network analysis (WGCNA) to construct gene networks in *P*. *trichocarpa*, resulting in 29 co-expression modules (Figure S3A) containing a variable number of genes ranging from 48 to 8884. The regulation networks between root tissue-specific genes (RTSGs, τ = 1) were established despite the paucity of tissue-specific TFs (TSTFs, τ = 1) (Figure S2). The magenta module had 376 genes, of which 96 were RTSGs (Tables S2 and S3). In this module, genes with high intramodular connectivity were considered intramodular hub genes (Tables S2 and S3). Among them, *Potri.013G151300*, encoding a fasciclin-like arabinogalactan protein, was found. Twelve root-specific TFs (RTSTFs, τ = 1) were linked to 332 genes, which encompasses 36 TFs. One of RTSTFs, *Potri.001G284300*, encoding MADS-box transcription factor was associated with 330 genes. In contrast, another RTSTF, *Potri.T125000*, that encodes a DNA binding domain in Myb protein, was predicted to have association with merely 13 genes. The aforementioned *Potri.T125000* and *Potri.001G284300*, along with *Potri.005G060900*, that encodes a sterol regulatory element-binding protein, were the only three non-syntenic genes among RTSTFs.

All genes in *P. tomentosa* were clustered into 20 co-expression modules (Figure S3B). The pink module encompasses 1243 genes, which can be siloed into three sets, including 310 constitutive genes, 96 TSGs, and 837 intermediate frequency expression genes. All 96 TSGs are root-specific genes (Tables S4 and S5). The hub gene of the pink module, *evm.TU.HIC_ASM_8.869* (*AT5G42650*, *Potri.004G148900*) encodes a cytochrome P450 enzyme. All RTSTFs are syntenic genes, among which 13 were found in the pink module and are in association with 48 TFs.

### 2.3. Identification of Conserved Motifs for Poplars in the Region of Genomes and Promoters

**Figure 2:**
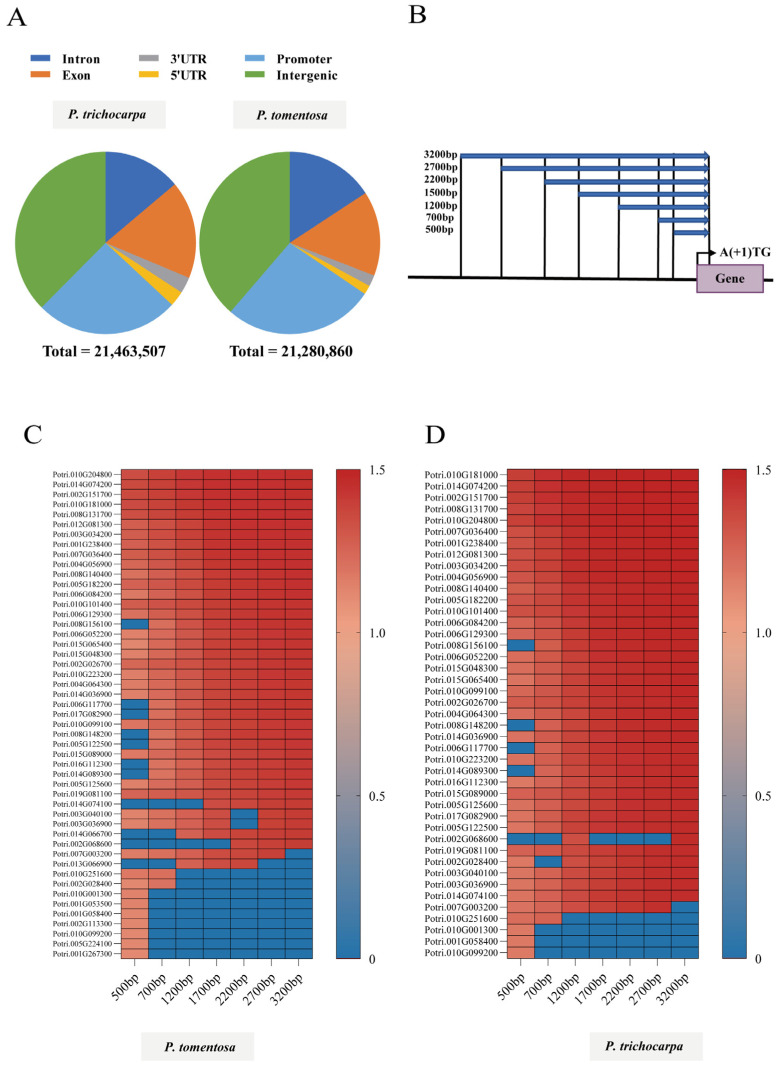
Identification of conserved TFBSs in genome and promoters of tissue-specific genes. (**A**) Pie chart representing the numbers of TFBSs that are detected in the genome of *Populus tomentosa* and *Populus trichocarpa*. The color indicates the region of genome. Promoters are defined as sequences up to 3000 bp upstream of the translation start codon (ATG), and intergenic represents downstream of the transcription termination site until the next promoter region. UTR, untranslated region. (**B**) Varying putative “promoter” sequence spaces were used to search for motifs conserved in different sets of genes. The schematic diagram indicates a representative gene with the ATG indicated. The potential regions include different lengths of sequences upstream the ATG (300 bp, 500 bp, 1.0 kb, 1.5 kb, 2.0 kb, 2.5 kb, 3.0 kb) as well as 200 bp downstream sequence. (**C**,**D**) Top 38 conserved motifs identified in each different lengths of promoters of tissue-specific genes. For each size of the promoter, the top 38 most conserved found using the promoter of *P. tomentosa* and *P. trichocarpa* space are shown (Frequency for conservation is indicated by color).

The rational design of synthetic promoters begins with identifying conserved CREs, which are non-coding DNA sequences recognized by specific TFs and responsible for the regulation of downstream genes [9]. Genomes were scanned using position weight matrices (PWMs) of 379 known TFs, revealing that the TFBSs were primarily localized within promoter sequences, coding regions, and intergenic regions, consistent with findings in *P. tomentosa* (Figure 2A).

To increase the likelihood of detecting conserved motifs, we first conducted a comprehensive search for putative promoters in diverse regions of the genome, spanning various lengths relative to the translation start codon (the first ATG) (Figure 2B). Putative promoters across all genes were compared for conserved TFBSs. By assessing the non-redundant top 38 (top 10%) most highly conserved motifs of 379 TFBSs in each search that used all sizes of promoters, we determined that the 2200 bp and 2700 bp regions in *P. tomentosa* had the same conserved TFBSs (Figure S4A), while this was the case in the 2700 bp and 3200 bp regions in *P. trichocarpa* (Figure S4B). And 1200 bp and 1700 bp regions had the same conserved TFBSs in both poplars. Forty-three conserved TFBSs were identified as shared by both *Poplulus* species (Figure S4C).

Next, we delved deeper into the variations in conserved TFBSs between potential promoters at the level of TSGs. TSG promoters of varying sizes were analyzed; as a result, 48 and 43 non-redundant TFBSs were identified from the top 38 most highly conserved motifs in *P. tomentosa* and *P. trichocarpa*, respectively (Figure 2C,D). It is intriguing that all of the most conserved TFBSs from TSG promoters in *P. trichocarpa* were also found in *P. tomentosa* despite the fact that their sizes differed widely. Specifically, in *P. trichocarpa*, the top 38 conserved TFBSs derived from 1700 bp promoters were identical to those obtained from 2200 bp and 2700 bp promoters (Figure 2D). In *P. tomentosa*, however, the top 38 (the top 10%) conserved TFBSs of the 48 non-redundant TFBSs were consistent in 2700 bp and 3200 bp promoter regions (Figure 2C).

Finally, the promoters of root tissue-specific genes (RTSGs, τ = 1) were investigated. There are 96 and 128 RTSGs in *P. tomentosa* and *P. trichocarpa*, respectively. We evaluated the 96 and 139 most conserved TFBSs from *P. tomentosa* and *P. trichocarpa*, respectively, which represent over 50% of the RTSGs (Figure S5A,B). From that, 95 non-redundant TFBSs were identified as the most conserved TFBSs of RTSGs (Figure S5C). Notably, the conserved TFBSs were not present in the 500 bp and 700 bp regions in proximity to the start codons. We generated a pool of 32 conserved TFBSs in root-specific promoters, which were removed from the “all genes” and TSG levels to permit more precise analysis (Figure S5D).

### 2.4. Generation of Models to Predict TFBS with High Importance Score for Tissue Specificity

**Figure 3:**
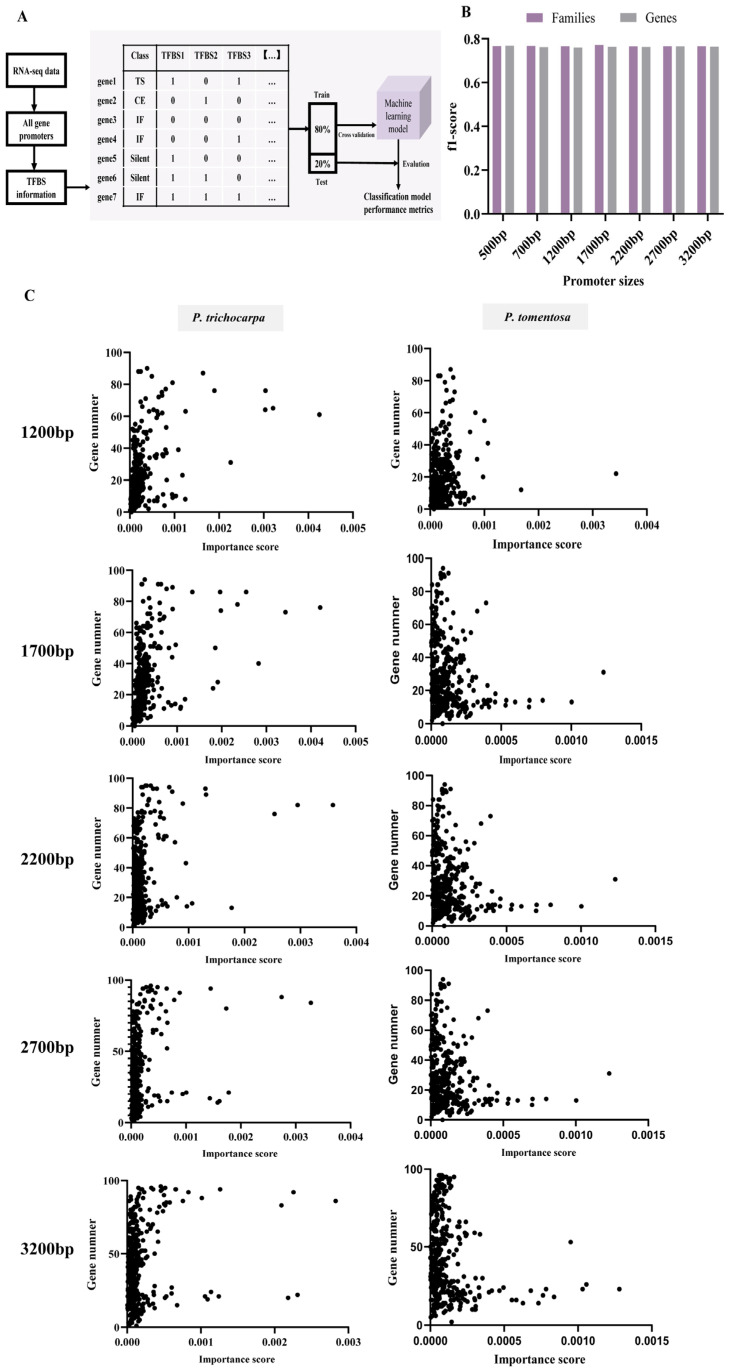
Training scheme and performance (f1-score) evaluation of different machine learning models predicting tissue-specific expression. (**A**) Training workflow and four training schemes. All genes are labeled as tissue-specific (TS), constitutive (CE), intermediate frequency (IF) and silent (Silent), respectively. (**B**) Performance comparison of models using the family name or TF genes as features and presence/absence data to train in all promoter spaces of *Populus tomentosa*. (**C**) Relationship between motif conservation level and feature importance score in 1200 bp, 1700 bp, 2200 bp, 2700 bp, and 3200 bp promoters. In *P*. *tomentosa* and *Populus trichocarpa*, all informative motifs were assessed for each set of promoters. Permutation-based feature importance score of TFBSs from random forest models ordered from least informative to most informative (*x*-axis). All TFBSs were assessed for each group of promoters. Motif conservation levels were determined by using motif occurrences in each gene of root tissue specific genes (RTSGs) sets (Gene number) and ordered from least significant to most significant (*y*-axis).

In this study, we sought to directly infer the activity of TFs that can best explain observed differential gene expression in different tissues in *Populus* by leveraging RNA-seq data and PWMs of TFs. For this purpose, we employed ML models in combination with several feature selection methods to determine the optimal parameters and training strategies (Figure 3A). Our pipeline entailed two primary phases. The initial phase involved the integration of genomic data sets, including the expression data from RNA-seq and the presence or absence of 379 TFBSs in both poplar genomes and promoters of varying sizes (Figure 2B). This process generated data sets for training, validating, and testing ML models. In the second phase, numerous classifications were performed using permutation-based feature selection methods. The input feature matrix for classification was constructed from binding site information for a list of differentially expressed genes, which were categorized by expression level and the τ value as described in the procedures (Figure 3A).

We aimed to generate and evaluate predictive models for gene expression by utilizing the feature matrix consisting of 379 TFBSs to classify gene expression and determine if the presence of TFBSs could be used to accurately predict tissue specificity. As is evident in Figure 3B and Table S6, moderate accuracy in predicting tissue specificity was achieved in *P. tomentosa* (micro-F1 score: 0.765–0.771) using the TFBS feature matrix. The spatially differential gene expression was best explained by the involvement of TFs from MIKC_MADS, TALE, BBR.BPC, ERF, Dof, GRAS, M.type_MADS, bHLH, BES1, and AP2 families, which were shown to have importance scores in the range of 1200–3200 bp (Table S7). In addition, we developed random forest models to identify TFs that significantly influenced the model performance in the promoter regions of *P. tomentosa*. There was, therefore, no significant variation in the micro-F1 score between the two types of features (families and genes) (Figure 3B).

Next, we developed predictive models for *P. trichocarpa* to find TFBSs with high scores as complements. As a result, 72 and 98 non-redundant TFBSs with high importance scores (top 50) were identified for *P. trichocarpa* and *P. tomentosa*, respectively, using the models for 1200–3200 bp region of RTSGs. However, we were not able to find any most conserved TFBSs in the 500–700 bp region. To assess whether the most highly conserved motifs are also the most predictive features, we compared the motif conservation ranks with the feature relevance of each motif in the predictive models for every RTSG promoter (Figure 3C, Table S8). Surprisingly, we found no evidence of a positive correlation between feature importance score and motif conservation rank. In other words, the model’s most conserved motif was not necessarily the most informative one, and vice versa (Figure 3C). Highly conserved TFBSs are frequently associated with housekeeping functions or fundamental cellular processes that are required across all tissues and conditions [30]. For example, motifs bound by basal transcription machinery components or general transcription factors would be expected to show high conservation but low tissue-specific predictive power. This is consistent with our observation that some of the most conserved motifs across the genome were filtered out when we focused specifically on root-specific genes (Figure S5D). This phenomenon aligns with studies showing that conserved noncoding sequences frequently mediate core regulatory functions rather than tissue-specific patterning [21].

### 2.5. Strategies for Designing Root-Specific Synthetic Promoters by Predicting TFBS with High Importance Score for Tissue Specificity

**Figure 4:**
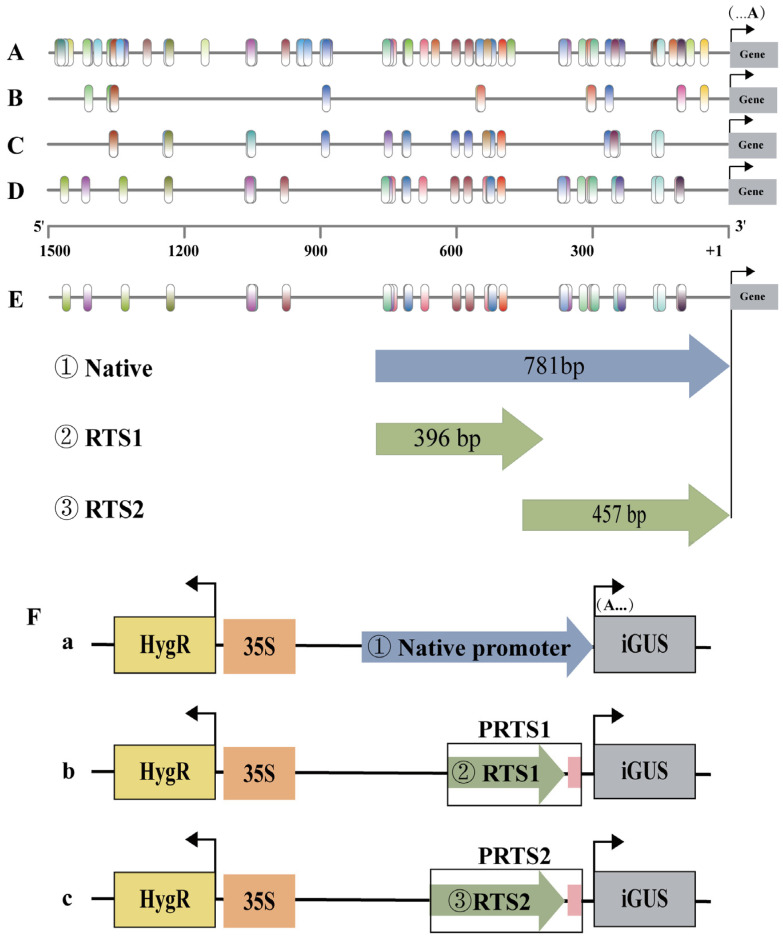
Analysis of transcription factor binding sites (TFBSs) of *PopRTS1* promoter and strategies for synthetic promoters. (**A**) Total of 146 TFBSs were found in the promoter of tissue-specific *PopRTS1*. The first base (A…, “…” signifies 3′ end) of the transcriptional start codon was taken as +1, and the region from −1500 to −1 relative to (A…) was for *PopRTS1* promoter. The TFBSS in promoters were represented by ellipses of various colors. (**B**) 53 most conserved in root tissue specific genes (RTSGs) remained in the promoter of *PopRTS1*. (**C**,**D**) Nonredundant top 50 TFBSs with high feature importance for *Populus trichocarpa* (**C**) and *Populus tomentosa* (**D**) remained in the promoter of *PopRTS1*, respectively. (**E**) Depending on the position of TFBSs, putative root-specific promoters are selected. −1~−781 bp is labeled as a native promoter, which was truncated into two domains (RTS1 and RTS2) with more informative TFBSs. The arrow indicates the direction in which putative promoters are regulated. (**F**) Binary vectors contained native and synthetic promoters (PRTS1 and PRTS2) fused to the β-glucuronidase (GUS) gene. The pink boxes represent 35Smini promoter. GUS represents the intron-containing GUS gene. HygR represents the hygromycin phosphotransferase gene that confers HygromycinB resistance in plants. The arrow represents the promoter regulation direction.

To validate the informative TFBSs identified in our previous analysis, we focused on a 1500-bp promoter region of an RTSTF, *Pop_A01G005994* (*PopRTS1*), which shares homology with *Potri.001G284300* that was most highly expressed of all RTSGs. As revealed by qRT-PCR analysis, *PopRTS1* displayed high level of expression in roots, but not in stems and leaves (Figure S6). Within the 1500-bp promoter region, we identified 146 TFBSs in association with 100 non-redundant TFs of 20 families (Figure 4A, Table S9). These TFBSs were primarily enriched in the ranges of −1250 bp~−1500 bp, −900 bp~1050 bp, −500 bp~−700 bp, and −50 bp~−400 bp regions (Figure 4A). Given that retaining only the 32 most conserved TFBSs in RTSGs (Figure 4B) made it difficult to identify meaningful CRMs, we selected 98 and 72 non-redundant TFBSs with high importance scores (top 50) for *P. tomentosa* and *P. trichocarpa* (Figure 4C,D), respectively, which allowed us to narrow down the enriched regions to −100 bp~−400 bp and −500 bp~−750 bp for both species. These findings imply that our predictive models for TFBSs are a valuable tool for identifying informative TFBSs within promoter regions and may help elucidate the regulatory processes of gene expression in *Populus* species.

In this study, we have developed a unique vector system that employs two reporter genes for the quantitative evaluation of specific promoter activity in plants. We merged the −396 bp~−781 bp (RTS1) and −1 bp~−457 bp (RTS2) domains within the *PopRTS1* promoter and the 35Smini::GUS (Figure 4E), generating synthetic promoters PRTS1 and PRTS2, respectively (Figure 4F). PRTS1 entails 21 TFBSs belonging to eight families, including MYB, MYB_related, Dof, HD-ZIP, C2H2, AP2, ERF, and MIKC_MADS, whereas PRTS2 entails 46 TFBSs belonging to 14 families, including C2H2, HD-ZIP, bHLH, Dof, ZF-HD, Trihelix, MYB, bZIP, NAC, MIKC_MADS, YABBY, G2-like, RAV, and AP2 (Table S9). Additionally, a native *PopRTS1* promoter sequence spanning −781 bp~−1 bp was also used to drive the GUS gene (Figure 4F). Both the native and synthetic promoter constructs were identical in vector architecture and backbone sequences, all containing a constitutively expressed 35S::HygR internal control cassette to facilitate selection of transgenic plants and monitor relative expression strength (Figure 4F).

### 2.6. Identification of Tissue Specificity for Native and Synthetic Promoters

**Figure 5:**
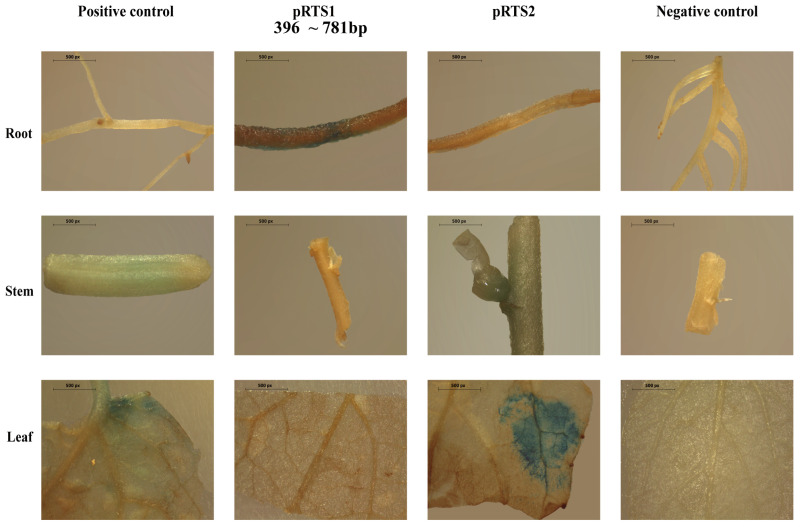
Tissue-specific activity for native and synthetic promoters. The histochemical assay for β-glucuronidase (GUS) activity in different tissues of poplars shows that the PRTS1 promoter was able to drive GUS expression in a root-specific manner. Poplar plants transiently expressing 35Smini-GUS and wild-type served as positive and negative controls, respectively.

Ten independent transgenic tobacco lines transformed with each of the constructs mentioned above were generated and used to assess the activities of their respective promoters using a fluorometric GUS assay. As shown in Figure S7, the 781-bp native promoter conferred significant root specificity. To validate the narrowed domain, we transfected poplar tissues with a frequency ranging from 51.3% to 57.6%, using CaMV 35Smini (modified pCAMBIA1301 vector) transformant and wild-type 84k as positive and negative controls, respectively. As a result, PRTS1 exhibited significant root-specific GUS expression, but PRTS2 abolished the GUS expression in the root (Figure 5). These findings indicate that the synthetic promoter PRTS1 could effectively drive root-specific expression, which can be attributed to a positive regulatory region spanning −396 bp to −781 bp.

### 2.7. Research Scheme to Develop Synthetic Promoters for Root-Specificity in Poplar

**Figure 6:**
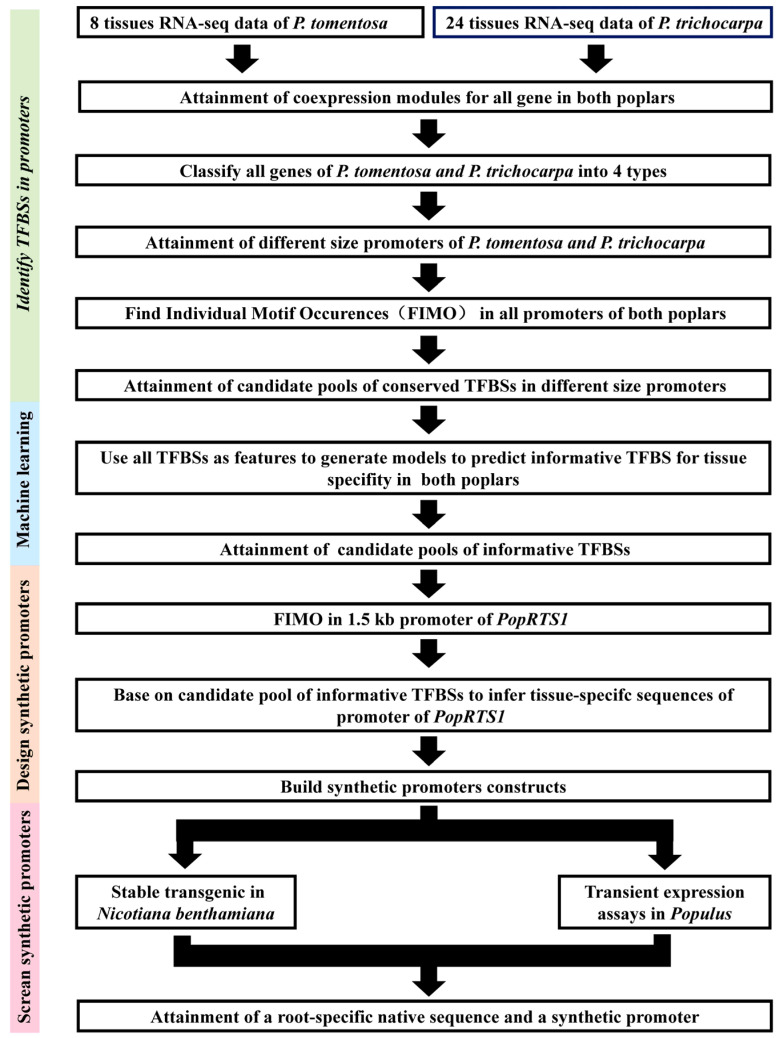
Research scheme to develop root synthetic promoter by machine learning in poplar.

As depicted in Figure 6, a root-specific synthetic promoter was designed utilizing ML, and synthesized by concatenating potential domains upstream of the CaMV 35S-46 core promoter sequence. This function of such a synthetic promoter was investigated by driving a GUS reporter gene.

Multifaceted approach was used to evaluate the TFBS motifs for designing synthetic promoters. Initially, *Populus* genes with tissue-specific, constitutive, silent, and intermediate frequency expression patterns were categorized in light of RNA-seq data across diverse tissues. Transcriptionally coregulated and functionally related genes were identified by establishing co-expression networks based on the expression data of 24 tissue transcriptomes from *P. trichocarpa* and eight from *P. tomentosa*. In the second step, significantly conserved motifs at various positions of putative promoters were identified using the FIMO algorithm. These conserved motifs were found to be present at three different levels, including all genes, tissue-specific genes, and root-specific genes in the two *Populus* species, resulting in six candidate pools of cis-acting elements, from which the most conserved non-redundant motifs from each promoter were chosen in light of various criteria. Following the removal of the TFBSs that were most conserved at the levels of all genes and tissue-specific genes (other than the root-specific), a pool of the conserved TFBS pool for root tissue-specific promoters was obtained (Figure S5D).

To develop predictive models of gene expression in poplars using TFBSs as a feature, which were categorized according to their expression patterns. The parameters and training strategies were optimized by ML models in conjunction with various feature selection approaches (Figure 3A). Based on the model performance score (f1-score) and promoter sizes, we found ten candidate pools of informative TFBSs for both *P. trichocarpa* and *P. tomentosa*, each comprising 50 TFBSs.

Two putative root-specific sequences were selected based on the frequency and position of the conserved and informative TFBSs in the promoter of *PopRTS1*, which served as the key elements of the synthetic promoter (Figure 4). Two synthetic promoters were ultimately evaluated by driving the GUS reporter gene in stably transformed tobacco and transient expression in *populus* (Figure 5).

## 3. Discussion

Recent advancements in synthetic biology hold the potential to revolutionize forestry research and development and usher in a new era of genetic improvements in forestry [31,32]. One of the key areas of application in forest tree breeding systems is trait enhancement, in particular, growth rate, wood properties, resistance to pests and diseases, and tolerance to abiotic stresses [33,34]. Gene editing, synthetic promoters, and transcription factors are among the most mature technologies in plant synthetic biology [25,35,36,37]. Synthetic promoters, in particular, have been employed to greatly expand the repertoire of regulatory tools for tuning gene expression in plants, providing researchers with greater control, precision, and flexibility in modulating gene expression [38,39,40]. The ability to design promoters with specific regulatory elements that respond to specific cues has greatly expanded the potential applications of synthetic biology in plant research and biotechnology. However, a major challenge in designing synthetic promoters is to identify tissue- and condition-specific CREs and CRMs, which are essential for driving gene expression in particular cell types, developmental stages, or under specific environmental conditions [8,30]. Identifying these regulatory elements and modules is critical for designing synthetic promoters with the desired spatiotemporal control over gene expression. In this study, we have identified a key regulatory module in governing root-specificity, by leveraging RNA-seq analysis and ML algorithms in *P*. *trichocarpa* and *P*. *tomentosa*, which were functionally validated in the poplar’s tissues. In addition, the tandem synthetic promoters, PRTS1 and PRTS2, were functionally validated by transient expression in poplars, highlighting the combinatory approach of transcriptomic analysis and ML as an effective method in identifying significant tissue-specific CRMs.

ML, particularly ensemble decision-tree models like random forests, has become a popular and powerful tool in plant synthetic biology research due to its ability to handle large and complex datasets as well as make accurate predictions based on the relationships between data and target values. Mean decrease in node impurity is an important metric for determining feature importance in decision tree-based models, which is conducive for researchers to identify the most relevant variables for making accurate predictions. The presence or absence of regulatory sequences can be informative features for classifying genes as up-regulated or down-regulated under certain conditions. When decision tree-based models, such as random forests, are used to classify genes based on their expression patterns, the presence or absence of these regulatory sequences can lead to a decrease in the mean node impurity, indicating that these features are informative for the classification task [41,42]. In this study, we classified genes into various categories, including silent, constitutive, tissue-specific, and intermediate frequency expression genes, based on their spatio-specificity, which is represented by the τ value (Figure 1C,D). By analyzing the presence or absence of TFBSs within these genes, we found that this feature was informative in classifying genes according to their τ values, as evidenced by a decrease in the mean node impurity (Figure 3A, Table S8). These findings demonstrate that the ability to identify informative features, such as TFBSs, and accurately classify genes based on their expression patterns can significantly advance our understanding of gene regulation and the functional implications of different expression profiles in various tissues.

Although our approach successfully identified informative TFBSs and categorized genes based on their tissue specificity, there are a few limitations that need to be acknowledged. The first limitation relates to our approach for classifying genes. We were able to identify TFBSs that contribute to the reduction in the mean node impurity and that are related to tissue specificity. However, the tissue specificity of which tissue is not informative due to the tendency of the mean decrease impurity score to inflate categorical features with a larger number of categories [21]. Furthermore, the number of tissue-specific genes in each tissue and the other three classes was not uniform (Figure 1C). This inherent biological imbalance and the complexity of transcriptional regulation likely contributed to the moderate predictive performance of our models (micro-F1 scores of 0.765–0.771), as solely relying on TFBS presence is insufficient to capture the full regulatory landscape. As a result, we classified tissue-specific genes from all tissues into a set (Figure 3A) and then selected the promoter of the root-specific gene for further analysis. The TFBSs are in the form of PWMs that are mapped to varying lengths of promoters. In this study, the presence or absence of CRMs consisting of informative TFBS sites in the putative promoter region may predict tissue specificity. However, we can only infer the domain, rather than the exact cis-element, for tissue specificity based on the assembly of the most informative TFBSs (Figure 4). The second limitation of our approach pertains to the types of information utilized in the model. Our current models were built using RNA-seq expression data with only PWMs of TFBSs. However, recent studies have demonstrated that incorporating epigenomic data such as unmethylated regions and accessible chromatin regions could enhance the identification of enriched motifs for training the model [21]. It is therefore expected that incorporating such data into future studies could improve the clustering of tissue-specific genes. Moreover, incorporating other regulatory information at the cell-type level resolution, such as TF interaction, DAP-seq and ChIP-seq data, chromatin accessibility, DNase I-seq, and single-cell RNA-seq, could further enhance the accuracy of our models [42,43,44,45,46]. The third limitation concerns the extent of experimental validation. In this study, functional validation of the identified CRMs and synthetic promoters was performed on a single root-specific gene, *PopRTS1*. We acknowledge that this limited scope raises questions about the generalizability of the approach to other genes or tissue types. However, it is important to emphasize that this work represents a proof-of-concept stage, aimed at establishing a feasible workflow for integrating machine learning with synthetic promoter design in poplar. Importantly, although validation was confined to one promoter, the informative TFBSs used to design it were derived from genome-wide analyses—including random forest models trained on all tissue-specific genes across two *Populus* species—and are therefore not gene-specific. The candidate CRM pools (e.g., the conserved root-specific TFBSs and top-ranked informative motifs) were identified from global regulatory patterns, meaning the screening strategy itself is inherently generalizable and can be readily applied to other genes of interest. Furthermore, the experimental design provided an internal validation of the model’s predictive power: the synthetic promoter PRTS1, designed based on regions enriched with high-importance TFBSs, successfully drove root-specific expression, while PRTS2, constructed from an adjacent region lacking these prioritized elements, failed to do so (Figure 5). This contrast demonstrates that the feature importance scores derived from our genome-wide model are functionally meaningful and capable of distinguishing functional from non-functional regions. Future work will extend this framework to other tissue-specific genes (e.g., xylem- or leaf-specific) to further validate the generalizability of the approach and expand the synthetic biology toolkit for poplar. Despite these limitations, our study exemplifies a cost-effective and efficient method for constructing tissue-specific synthetic promoters.

Putative intergenic CRMs are often conserved across species and under evolutionary constraint, indicating their functional importance [47,48,49]. Comparative genomic approaches have been employed to identify these conserved noncoding sequences (CNS) and CRMs by comparing the genomes of related species and identifying regions of conservation that are likely to have essential biological functions [30,50]. To effectively identify CREs and CRMs, researchers have utilized ensemble approaches that combine the predictions from multiple de novo motif-finding algorithms. These methods can improve the accuracy and sensitivity of motif discovery by leveraging the strengths of each individual algorithm while compensating for their weaknesses [51,52,53,54]. By integrating different algorithms, ensemble approaches can identify a more comprehensive set of motifs and provide a better understanding of the regulatory landscape [53,54]. The *PopRTS1* promoter contains numerous CREs within its 1500 bp region (Figure 4A), and previous studies have often divided it into 5–8 sections for functional validation [40,55,56,57]. However, this approach can be time- and labor-intensive, particularly in plants with long growth periods, such as woody plants. To circumvent this challenge, we used PWMs of 379 TFBSs to scan the genome and promoters of TSGs and RTSGs. This allowed us to identify pools of conserved TFBSs at the root-specific gene level (Figure 6 and Figure S5), which were further refined using ML to produce pools of informative TFBSs (Table S8). Putative CRMs were then identified as regions containing more TFBSs from the pools of informative TFBSs. Importantly, we prioritized the pools of informative TFBSs when identifying root-specific CRMs. For instance, although the PWM of the MIKC_MADS family (Potri.014G074100) was found in RTS2, the corresponding TF (Potri.014G074100) was not present in the pool of the informative TFBSs, leading to a lack of root specificity in PRTS2. By focusing on informative TFBSs, we were able to streamline the identification of root-specific CRMs and reduce the time and effort required for functional validation.

Intensive efforts to explore inducible or constitutive CREs have led to the development of synthetic promoters with tailored strengths and specificities. This is achieved by truncating CRMs into smaller sections and combining them with multiple CRE sequences, copy numbers, and spacing arrangements [25]. Synthetic promoters offer a customizable approach to gene regulation, allowing for more precise control of gene expression in various applications, including plant biotechnology and genetic engineering. One common strategy is to use synthetic regions upstream of a core promoter sequence, such as the 46 bp CaMv 35S promoter. This well-characterized minimal promoter has been extensively used in basic gene construct design due to its strong constitutive activity in a wide range of plant species. Recently, researchers have been leveraging CREs from various plant pathogens to construct minimal synthetic promoters with constitutive responses. By arranging these CREs in different positions relative to the TATA box area, they have been able to create synthetic promoter with unique properties [6]. Previous research has indicated that tissue-specific functions are largely regulated independently of TF expression, with tissue specificity being mediated by tissue-specific regulatory networks [58]. To investigate this further, WGCNA was used to attempt to identify tissue-specific TF networks of cooperations for root tissue in poplars (Tables S2–S5). Although numerous TF networks were identified in each tissue, and a single TF was found to be linked to multiple other TFs, tissue-specific networks of TFBSs could not be isolated in each tissue. This suggests that the regulatory mechanisms of tissue specificity may be more complex than previously anticipated, potentially involving additional factors or interactions. Despite these limitations, the findings provide a valuable starting point for future research aimed at understanding the molecular mechanisms that control root growth. By continuing to investigate and characterize the molecular components and interactions within these networks, research can gain a better understanding of the regulatory processes governing tissue-specific functions.

The relatively short length of CREs within CRMs and the higher sequence variability of flanking sequences surrounding CREs have been observed in previous studies [50]. To maintain the cooperative DNA-binding achieved through various mechanisms and by both proteins and DNA serving as scaffolds for TF binding (Figure 4E,F), all TFBSs were retained along with their native flanking sequences. While the optimization of CRM size was not attempted in this study, it may be a desirable step in future research. Other factors, such as the optimization of flanking sequences [56], the copy number of cis-elements [59], and the spacing between CREs and the core promoter [2,60], are also crucial for enhancing the strength and specificity of synthetic promoters.

Putative CRMs are typically cloned upstream or downstream of reporter genes, driven by either a minimal promoter or an enhancer, and their functions are evaluated by examining the activation or silencing status of the reporter genes through transient or transgenic assays [61,62]. Reporter gene activity is usually assessed by three methods. The first one is the transient transfection of protoplasts, which allows for rapid testing of gene activation or silencing due to the putative CRM. The second method involves transient gene expression using *Agrobacterium* infiltration into *Nicotiana benthamiana* leaves [7,63,64]. The third method involves the stable transformation of whole plants, typically using *Agrobacterium*-mediated transformation or particle bombardment. In this approach, transgenic plants containing the putative CRM and reporter genes are generated, and the reporter gene expression is analyzed in the stable transgenic lines [9]. The transient nature of the former two methods may not accurately represent the stable expression patterns of the gene of interest in the whole plant, and hence they are ineffective for screening tissue-specific synthetic promoters [9,65,66], and the stable transformation outlined in the latter method is laborious and time-consuming. The development of a simple, rapid, and efficient root transformation system has greatly facilitated the functional characterization of genes in various plant species. One such system uses *Agrobacterium rhizogenes* to generate transformed roots, known as hairy roots, which have been successfully developed for an eclectic range of medicinal and woody plant species [67,68]. This system enables researchers to study gene function in the context of transformed roots, providing insights into root-specific gene expression and function. PRTS2 might harbor active silencer or repressor elements that actively suppress transcription in roots. Negative regulatory elements play important roles in restricting gene expression to specific tissues in plants [69]. Notably, among the TFBS families identified in PRTS2, several—including ERF, NAC, and certain bHLH subfamilies—include members known to function as transcriptional repressors [70,71,72]. The presence of binding sites for these repressor families could potentially recruit negative regulators that override any weak activating signals present in this region. While histochemical GUS staining provided clear visualization of the tissue-specific activity of the synthetic promoter PRTS1 (Figure 5), we acknowledge the absence of a quantitative measurement, such as a MUG (4-methylumbelliferyl-β-D-glucuronide) fluorometric assay or qRT-PCR analysis of GUS transcript levels. Our current study primarily focused on establishing the spatial pattern of promoter activity—specifically, verifying root-specificity—for which qualitative staining is well-suited and widely accepted in plant biology. Nevertheless, quantitative data would offer a more precise assessment of promoter strength and facilitate comparisons with other synthetic constructs. Although we indirectly validated the activity of the native promoter through qRT-PCR analysis of the endogenous *PopRTS1* gene (Figure S6), future work should incorporate MUG assays or GUS transcript quantification to precisely determine the activity of synthetic promoters. Moreover, an extremely simple cut–dip–budding (CDB) delivery system has been developed using *A*. *rhizogene* to inoculate explants [67]. In this method, plant explants are cut and dipped into an A. rhizogene suspension, followed by the induction of root suckering to generate transformed buds. This CDB system offers a straightforward, efficient, and cost-effective approach to producing transformed roots for functional characterization.

In our study, we employed *Agrobacterium* infiltration as a method to deliver putative CRMs into multiple tissues in poplars, utilizing a reporter gene with an intron to minimize false positives that result from *Agrobacterium* infection. This approach allows for the efficient validation of synthetic promoters in a relatively short time frame of 3–4 days. *Agrobacterium* infiltration has been widely used to examine gene expression due to its simplicity and versatility [73]. Infiltration into leaves is generally easier compared to stems or roots; however, our modified protocol enables the simultaneous analysis of leaf, stem, and root tissues, offering a more comprehensive view of gene expression patterns across different plant tissues. For example, when a synthetic promoter exhibits root-specific expression, the root can be easily stained, as illustrated in Figure 5. This visualization method provides a convenient way to assess the activity of the synthetic promoter in the root tissues and determine its functional relevance.

Despite these advantages, we acknowledge that the transient expression system has inherent limitations regarding statistical rigor. The results from poplar tissues were reported as transformation frequencies (51.3% to 57.6%) based on multiple independent infiltration experiments, rather than as outcomes from a defined number of independent transgenic lines. While transient assays are widely accepted for rapid functional screening in plants, transient expression is inherently more variable than stable transformation, and the absence of a clearly specified sample size for this component limits the statistical rigor of the poplar validation data. Without detailed information on the number of biological replicates or independent experiments, it is difficult to fully assess the reproducibility and robustness of the results. Nevertheless, the consistent outcomes across multiple infiltration experiments support the conclusions drawn from the tobacco stable transformation data, where 10 independent lines per construct exhibited 100% concordance in their tissue-specific patterns. Future work should include clearly defined biological replicates for transient assays and incorporate quantitative measurements to complement the qualitative spatial expression data presented in this proof-of-concept study.

## 4. Materials and Methods

### 4.1. Plant Materials and Growth Conditions

All plants used in this research were established and maintained in a greenhouse at Beijing Forestry University (Beijing, China). One-month-old 84k clones were cultivated in glass bottles with inner dimensions of 11 cm in height and 7 cm in diameter, containing about 60 mL ½ MS (Murashige and Skoog, Sigma-Aldrich, St. Louis, MO, USA) medium supplemented with 0.05 mg/L IBA (Biorigin, Beijing, China), 0.05 mg/L NAA (Biorigin, Beijing, China), 7 g/L Phytoagar (Sigma-Aldrich, St. Louis, MO, USA), and 20 g/L sucrose (Biorigin, Beijing, China), with pH value being adjusted to 5.8–6.0.

One-month-old *Nicotiana tabacum* clones were maintained on rooting media (½MS (Murashige and Skoog, Sigma-Aldrich, St. Louis, MO, USA), 20 g/L sucrose (Biorigin, Beijing, China) and 7 g/L agar (Biorigin, Beijing, China), pH 5.8–6.0). The plant clones were cultivated under a light intensity of 70 µmol/m2/s provided by 14 W Coolwhite fluorescence tubes UH-BLDG12180-01 (Unihero, Guangzhou, China), at a temperature of 25 ± 2 °C, 50% ± 1% relative humidity, and a 16/8 h day/night regime in a climate-controlled growth room. Regenerated plants were propagated every 6 to 8 weeks.

### 4.2. DNA, RNA Purification and RNA-Sequencing Analysis

Fresh leaves were collected from one-month-old 84k clones and immediately used for total genomic DNA isolation according to the manufacturer’s protocol of the DNAsecure Plant Kit (Qiagen, Shanghai, China, DP320). The RNAs of each tissue were extracted by following the instruction of RNAprep Pure Plant Kit (Qiagen, Shanghai, China, DP432).

### 4.3. RNA-Seq Analysis and Gene Expression

Samples of various tissues were collected from *P*. *trichocarpa*, including emerging young leaf, fully expanded leaf, senescent leaf on both long and short branches, shoot apex, root, mature and immature xylem, cambium, phloem, bark, petiole, pistil, and stamen [74]. From *P*. *tomentosa*, RNAs were purified from old leaf, shoot apex, root, mature and immature xylem, cambium, phloem, and bark [13]. The *P*. *trichocarpa*_v3.0 genome (Accession ID: AARH04000000) produced by the National Center for Biotechnology Information (NCBI) [74] was used as the reference genome.

### 4.4. Measurement of Tissue Specificity

To analyze the tissue specificity of gene expression, the tissue specificity index τ was calculated as follows:

$$\tau =\frac{\sum_{i=1}^{n}\left(1-{\hat{x}}_{i}\right)}{n-1};{\hat{x}}_{i}=\frac{x_{i}}{\underset{1\leq i\leq n}{\max}\left(x_{i}\right)}$$

Here, n represents the number of tissues and $x_{i}$ represents the average expression level of the gene in tissue i, and ${\hat{x}}_{i}$ indicates the expression profile component normalized by the maximal component value. The index ranges from 0 to 1, where 0 signifies that a gene’s expression is identical in all tissues, and 1 indicates tissue-specific expression.

### 4.5. Weighted Gene Co-Expression Network Analysis of the Tissue-Specific Genes

The WGCNA was performed based on the expression levels of 34,106 genes in *P*. *tomentosa* and 41,335 genes in *P*. *trichocarpa*, using the WGCNA package in R 3.6.3 [75]. The correlations between the modules were represented with R values. The processing of WGCNA data is described in Supplementary Method S1.

In *P. trichocarpa* and *P. tomentosa*, the soft-thresholding powers from 1 to 30 were calculated using scale-free topology criteria, while a power of 28/24 was used in module identification. The minimodule size was set to 50, and the merge cut height was set to 0.28.

### 4.6. Characterization of Genes with Distinct Expression

According to the expression of annotated genes in the *P. tomentosa* genome, the genes not expressed were identified as “silent”. The value of τ was used to identify tissue-specific genes. The genes with a τ value equals to 1.00 were referred to as “tissue-specific”, while those with a τ value less than 0.85 (CV < 0.30) were designated as having “constitutive expression”. The remaining genes were designated as having an “intermediate frequency of expression”.

### 4.7. Cis-Elements, Conserved Motifs, and Chromosomal Mapping Analysis

A total of 379 known TF binding motifs from *P. trichocarpa* were obtained from PlantTFDB v5.0 [76]. Enriched motifs were identified using FIMO (version 5.4.1). Gene structures and conserved motifs were visualized using Tbtools (version 1.0).

### 4.8. Training of Machine Learning Models to Predict Tissue-Specific Motifs

To predict the motifs associated with tissue-specific regulation, families of TFBSs and genes of TFBSs were investigated. For each feature set, the effect of different search spaces, including different promoter sizes (0.5, 0.7, 1.2, 1.7, 2.2, 2.7, and 3.2 kb) was investigated.

To train random forest models to predict tissue-specific motifs, distinct sets of promoters were used. Each model was trained by first splitting the data into a training set consisting of 80% and a test set consisting of the remaining 20%. Using 10-fold cross validation, the training was conducted solely on the training set. The grid search algorithm (“tune_grid” function) in R’s tidymodels (version 0.1.3) was used to find the optimal values for the model hyperparameters (the number of predictors, the number of trees, and the minimum number of data points in a node). Subsequently, the performance metrics (F1 score) of 20 models were collected. The R (version 4.1.2) package “ranger” (version 0.16.0) was used to derive permutation-based feature importance scores for each input motif from trained models.

### 4.9. Construction of Fusion Plasmid with GUS Reporter Gene

Through a BLAST (version 2.12) search with the protein sequences of *P*. *trichocarpa* annotation of the tissue-specific gene to *P. tomentosa* and 84k genome assembly, about 781 kb upstream promoter sequence from ATG of the *Pop_A01G005994* were collected from the 84k genome. The primer RP (5′-CACAATCTTCCCTCTCCCCAT-3′) and FP (5′-CTTTCAGTAGCCATAATCAAC-3′) were designed based on the sequences of *Pop_A01G005994* promoter from the 84k genome to construct clone plasmids using the pEASY^®^-Blunt Cloning Kit (TransGen Biotech, Beijing, China). After sequencing verification, the primers pFR (5′-aagcttggctgcaggCTTTCAGTAGCCATAATCAACAATC-3′) and pRP (5′-aggatagtgggattgCTCGCTATTCTGGGATTTTTACACA-3′) were designed on the website (https://soft.transgen.com.cn/index.php, 28 October 2021)), with appropriate restriction sites SalI and BamHI at the ends, respectively. PCR products were fractionated by electrophoresis in a 1% agarose gel, from which dimerized products were purified by TIANgel Midi Purification Kit (Qiagen, Shanghai, China, DP209). The PCR product was subsequently cloned into the designated restriction sites of the pCAMBIA1301 vector that was obtained from the MiaoLing Plasmid Sharing Platform, using the pEASY^®^-Basic Seamless Cloning and Assembly Kit (Transgen Biotech, Beijing, China).

Synthetic promoter fragments were designed as illustrated in Figure 4F. The PRTS1 and PRTS2 were generated by reannealing two complementary single-stranded oligonucleotides containing a 5′ overhang of SalI digestion site and a BamHI digestion site at 3′ end. All promoter-construct DNA sequences and ligation conditions were verified by Sanger sequencing analysis (Sangon Biotech, Shanghai, China).

### 4.10. Stable Transformation and Transient Agroinfiltration Assay in Plants

The fusion plasmids were introduced into *Agrobacterium tumefaciens* strain GV3101 by the heat shock method [77]. *A*. *tumefaciens* cells harboring the construct were inoculated in a YEB medium that entails 5 g/L beef extract (Thermo Scientific™ Oxoid, Basingstoke, UK), 1 g/L yeast extract (Thermo Scientific™ Oxoid, Basingstoke, UK), 5 g/L peptone (Thermo Scientific™ Oxoid, Basingstoke, UK), 5 g/L sucrose (Biorigin, Beijing, China), and 0.5 g/L MgCl_2_ (Sigma-Aldrich, St. Louis, MO, USA), supplemented with 50 mg/L rifampicin (Biorigin, Beijing, China) and 50 mg/L kanamycin (Biorigin, Beijing, China). The culture was incubated at 28 °C for 12 h with shaking. Following centrifugation at 6000× *g* for 10 min, the pellet was re-suspended in the pre-infiltration liquid MS medium supplemented with 200 μM acetosyringone (Biorigin, Beijing, China) at a density of 1.0–1.2 OD600 For transient assay, 84k seedlings were dipped into the infiltration medium and infiltrated under vacuum in a degassing chamber at 60 mBar for 1 h. Gloves were changed after the infiltration of each construct to prevent contamination. 84k seedlings were cultured under the same conditions (25 °C, 8 h under light and 16 h in darkness) for 2 d after injection.

For stable expression, the *A. tumefaciens* cells harboring the vector were infiltrated into the abaxial side of the third and fourth leaves of *N. tabacum* by pushing the syringe without a needle, as previously described [78]. Tobacco leaf discs were transformed essentially according to the method of Horsch et al. [79]. Transgenic *N. benthamiana* plants were screened on MS plates containing 1 mg/L 6-BA (Biorigin, Beijing, China), 0.1 mg/L IBA (Biorigin, Beijing, China), 30 g/L sucrose (Biorigin, Beijing, China), 7 g/L Phytoagar (Sigma-Aldrich, St. Louis, MO, USA), 50 mg/L kanamycin (Biorigin, Beijing, China) and 200 mg/L Timentin (Biorigin, Beijing, China).

### 4.11. Quantitative PCR Analysis

To validate the identified root-specific promoters, quantitative reverse transcriptase PCR (qRT-PCR) analysis of gene expression driven by the native promoter was performed. The primer sets used for validation are listed in Table S1. The qRT-PCR was performed using the ABI StepOne Plus instrument (Applied Biosystems, Foster City, CA, USA). The PCR amplification program was as follows: initial hold stage at 95 °C for 30 s; 40 cycles of 95 °C for 5 s, and 60 °C for 35 s, and a final curve stage at 95 °C for 15 s; 60 °C for 1 min and 95◦C for 15 s. Sample cycle threshold (Ct) values were determined and standardized relative to the endogenous control gene, and the 2^−∆∆CT^ method was used to calculate relative changes in gene expression based on the qRT-PCR data according to previous research [80].

The expression patterns of *Pop_A01G005994* (*PopRTS1*) in root, stem, and leaf were also validated by qRT-PCR analysis using the primer sets (qPopRSF/R) listed in Table S1. A melting curve was used to check the specificity of each amplified fragment. For all reactions, triplicate technical and biological repetitions were performed for each individual with actin (*NM_001325686*) or 18s (*Potri.010G138100*) used as the internal control.

### 4.12. Histochemical GUS Assay in Transgenic Plants

Transgenic seedlings were initially immersed in precooled 90% acetone (TGREAG, Beijing, China) for 20–30 min. Following proper cleaning, the fixed seedlings were stained in GUS staining buffer comprising 50 mM sodium phosphate (MACLIN, Shanghai, China), 2 mM cyclohexyl ammonium salt (MACLIN, Shanghai, China), 0.5 mM K_3_Fe(CN)_6_ (MACLIN, Shanghai, China), and 0.5 mM K_4_Fe(CN)_6_ (Coolaber, Beijing, China), pH 7.2, supplemented with 0.5 mM X-Gluc (Coolaber, Beijing, China) as a substrate for β- glucuronidase (GUS) activity, and incubated at 37 °C for 8 h in the dark to allow GUS staining to occur. Then, the seedlings were de-stained using a series of ethanol (TGREAG, Beijing, China) washes (20%, 50%, 70%, 90%) for 20–30 min each to remove chlorophyll and enhance the visibility of GUS staining. Finally, all the stained seedlings and tissues were observed under a Leica EZ4 HD stereomicroscope (Leica, Nussloch, Germany) or a microscope fitted with the SunGrant SOPTOP OD400 digital camera (SunGrant, Suzhou, China) [81].

## 5. Conclusions

In summary, the identification of CREs and CRMs is crucial for achieving precise expression patterns through the use of synthetic promoters in transgenic plants, a process that can be facilitated by ML methods. In this study, we evaluated transcriptome data from poplar tissues to determine tissue-specific TFBSs with tissue specificity. Subsequently, we used this information to design synthetic promoters that were exclusively active in the root tissue. Rapid infiltration techniques and GUS staining was employed to verify the activity of these promoters, ultimately leading to the discovery of synthetic root-specific promoter PRTS1. With implications for target gene expression systems in plant biotechnology, this study presents a systematic strategy for identifying tissue-specific CRMs and gives a practical method for screening and generating functional synthetic promoters in polar.

## Data Availability

The transcriptome expression data (three biological replicates per group) are available in the National Center for Biotechnology Information SRA database under accession numbers PRJNA521819, PRJNA521855, PRJNA522886, PRJNA522891, PRJNA357670, SRP141094, SRP073689, and SRP060593.

## Supplementary Materials

The following supporting information can be downloaded at: https://www.mdpi.com/article/10.3390/ijms27062540/s1.
