# Supplementary material for: Design and Testing of Root-Specific Synthetic Promoters by Machine Learning
Source: Int J Mol Sci. 2026 Mar 10;27(6):2540. doi: 10.3390/ijms27062540 (PMC13026494; doi:10.3390/ijms27062540)
Supplement: Supplementary file 1 [file ijms-27-02540-s001.zip › Supporting Information Methods S1 and Supplementary Figures S1–S7.pdf]

Supplementary Material

1、Supplementary Method

Supplementary Method S1 The processing of WGCNA data (Double-click to open)

```
rm(list = ls())
Expr1 <- read.table("tissuespecific.txt", header = TRUE)
Expr2 <- log(Expr1+1, 2)
dim(Expr2)

library(WGCNA) # version 1.63
library(flashClust)
library(reshape2)
library(stringr)
library(BiocParallel)
options(stringsAsFactors = FALSE)
enableWGCNAThreads()# mutiple Threads
register(MulticoreParam(60))
allowWGCNAThreads(nThreads = 60)

corType = "pearson" # correlation method,another correlation type is
“bicor”
maxPOutliers = 0.05
robustY = FALSE  # Dealing with binary data
Expr_t <- as.data.frame(t(Expr2))


sampleTree <- hclust(dist(Expr_t), method = "average")
par(mar = c(0,4,2,0))
pdf(file = "sampleTree.pdf", width = 12, height = 9)
plot(sampleTree, main = "Sample clustering to detect outliers",
sub="", xlab="")
dev.off()
# call outlier samples
clust <- cutreeStatic(sampleTree, cutHeight = 260, minSize = 8)
rownames(Expr_t)[clust==0]
keepSamples <- (clust != 0)
Expr3 <- Expr_t[keepSamples, ]
dim(Expr3)
geneNames <- colnames(Expr3)

powers <- c(seq(1, 10, by=1), seq(12, 30, by=2))
type ="unsigned"
sft = pickSoftThreshold(Expr3, powerVector=powers, networkType=type,
verbose=5)
sizeGrWindow (9,5)
par(mfrow= c(1,2))
cex1=0.9
pdf("wgcna_soft.thresholding.pdf")
```

2、Supplementary Figures and Tables

Supplementary Figure S1. Histogram of frequency for all genes in a range of τ and CV.

Supplementary Figure S2. The number of TFs (τ=1) that are detected in each tissue.

Supplementary Figure S3. Construction of the WGCNA model.

Supplementary Figure S4. Identification of conserved TFBSs in all genes.

Supplementary Figure S5. Identification of conserved TFBSs in promoters of RTSGs.

Supplementary Figure S6. Expression analysis of PopRTS1 by qRT-PCR.

Supplementary Figure S7. Expression analysis of *GUS* driven by native promoter.

Supplementary Table S1 The primer sets used in this study.

Supplementary Table S2 The expression of magenta module in *Populus trichocarpa*.

Supplementary Table S3 The interaction of magenta module in *Populus trichocarpa*.

Supplementary Table S4 The expression of the pink module in *Populus tomentosa*.

Supplementary Table S5 The interaction of the pink module in *Populus tomentosa*.

Supplementary Table S6 The performance of the predictive models by machine learning for *Populus trichocarpa* and *Populus tomentosa*.

Supplementary Table S7 Non-redundant families of TFBSs with high importance score (top 5) for *Populus tomentosa*.

Supplementary Table S8 Non-redundant TFBSs with high importance score (top 50) for *Populus trichocarpa and Populus tomentosa*.

Supplementary Table S9 FIMO results for the promoter of *PopRTS1*

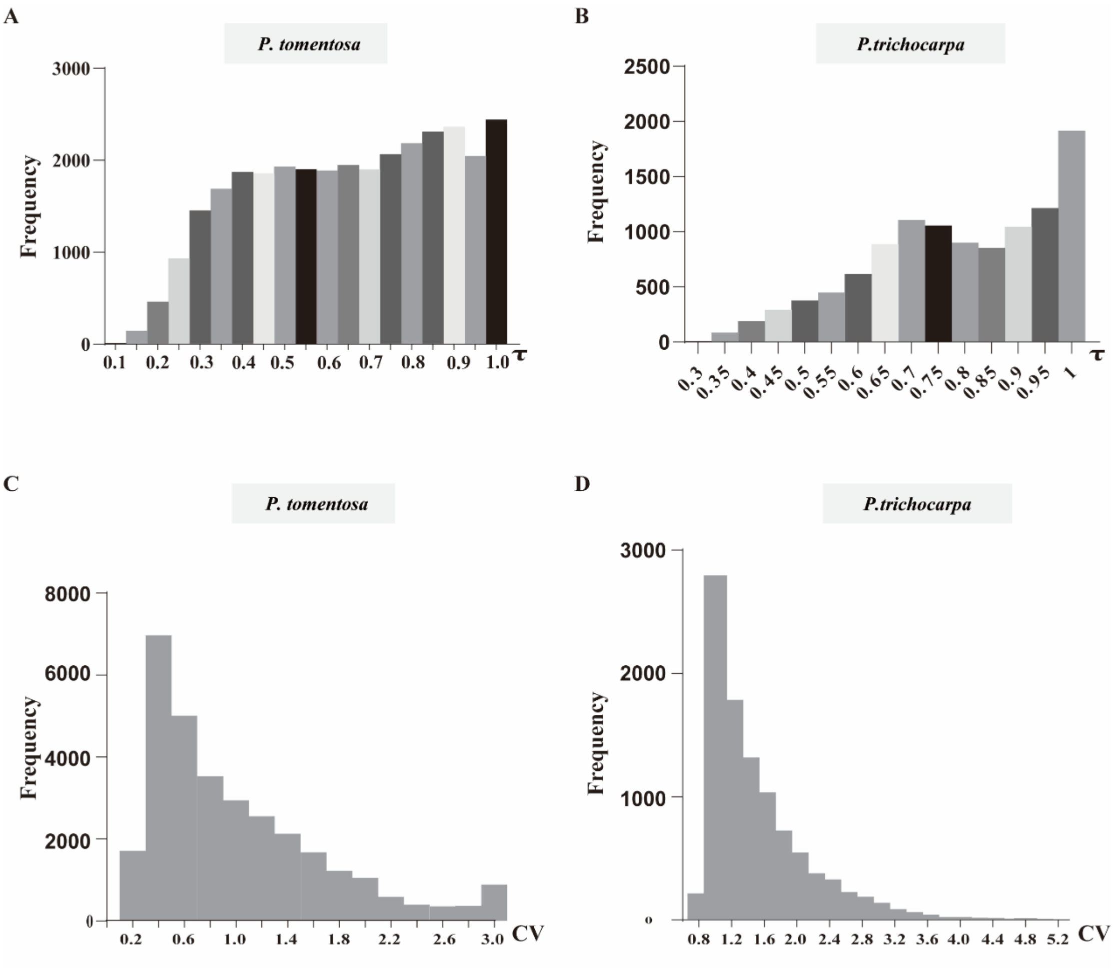

Supplementary Figure S1. Histogram of frequency for all genes in a range of τ and CV.

(A and B) Histogram shows the number of frequencies that fall within a specified range of τ in *P. tomentosa* and *P. trichocarpa.*

(C and D) Histogram shows the number of genes that fall within a specified range of CV in *P. tomentosa* and *P. trichocarpa.*

A

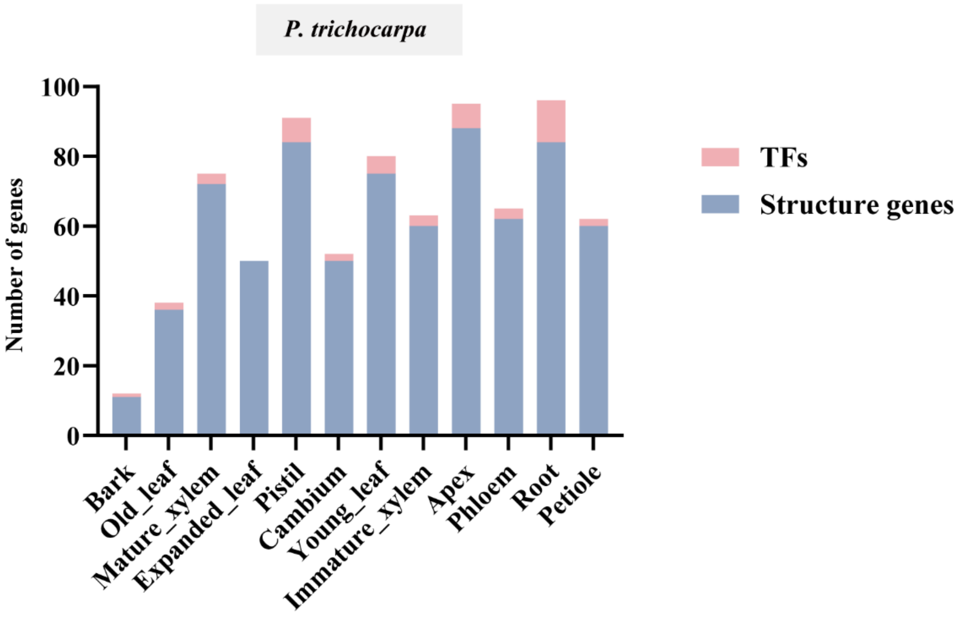

B

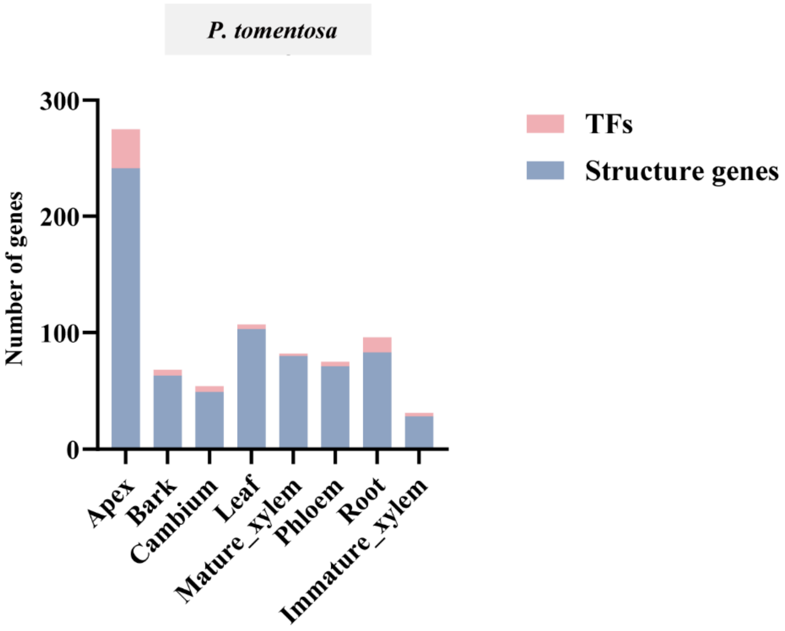

Supplementary Figure S2. The number of TFs (τ=1) that are detected in each tissue.

(A and B) The number of TFs that are detected in each tissue is shown. The color indicates the function of the gene as TFs (pink) or Structural genes (light purple) in *P. trichocarpa and P. tomentosa*. MX: mature xylem, EL: expanded leaf, IMX: immature xylem, YL: young leaf, OL: old leaf.

A

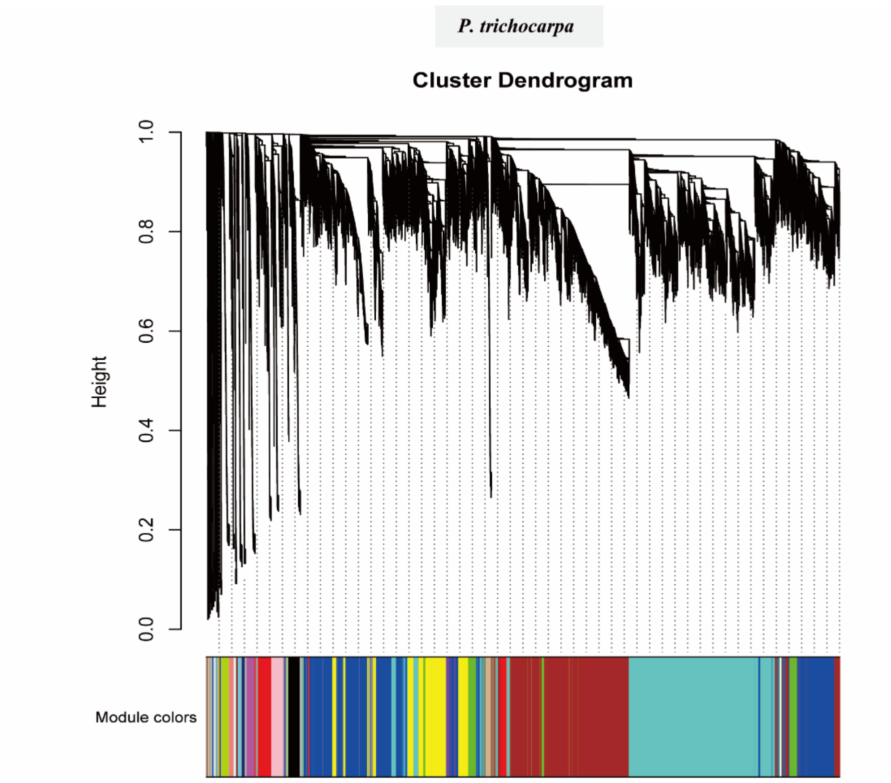

B

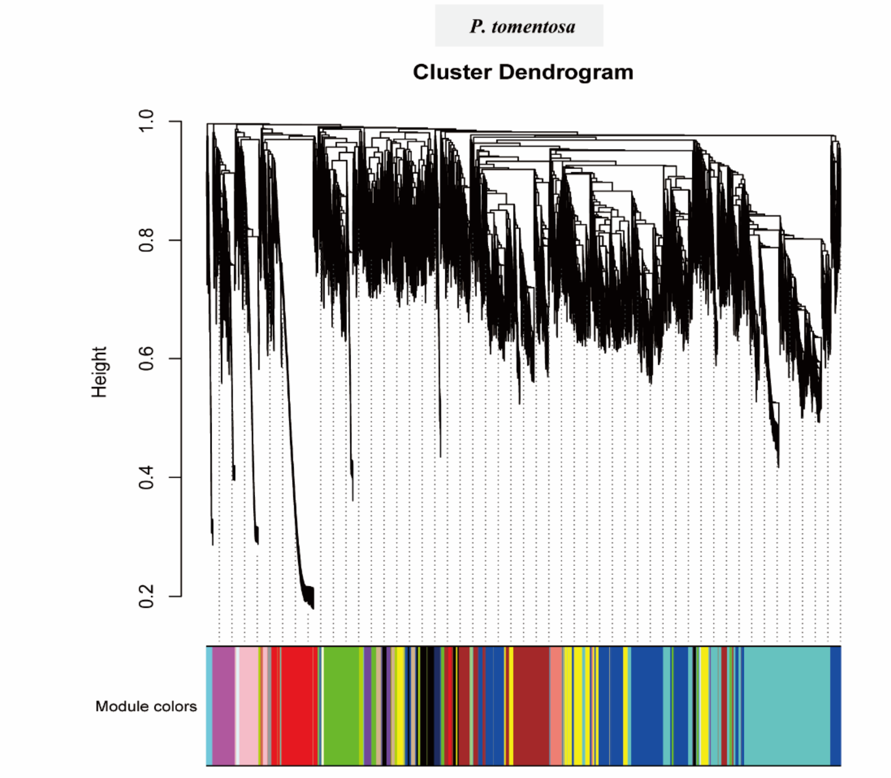

Supplementary Figure S3. Construction of the WGCNA model.

(A) Coexpression modules identified by WGCNA. The major tree branches represent 29 modules for *P. trichocarpa*.

(B) Coexpression modules identified by WGCNA. The major tree branches represent 20 modules for *P. tomentosa*.

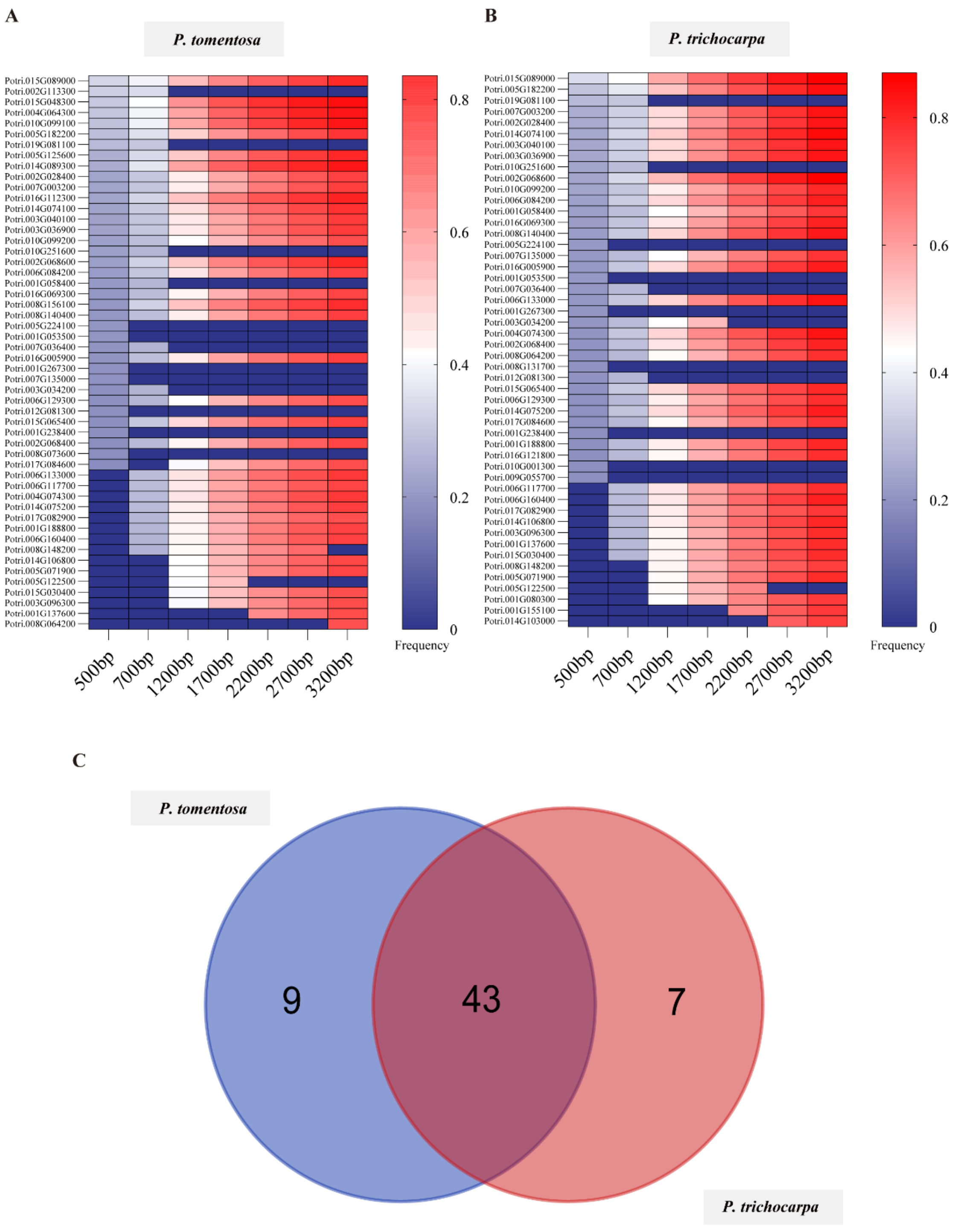

Supplementary Figure S4. Identification of conserved TFBSs in all genes.

(A and B) Nonredundant top 38 conserved motifs identified in each different lengths of promoters. For each size of the promoter, the top 38 most conserved found using the promoter of *P. tomentosa* and P. trichocarpa space are shown (P-value for conservation is indicated by color).

(C) The overlap of top 38 conserved motifs of *P. trichocarpa* and *P. tomentosa*.

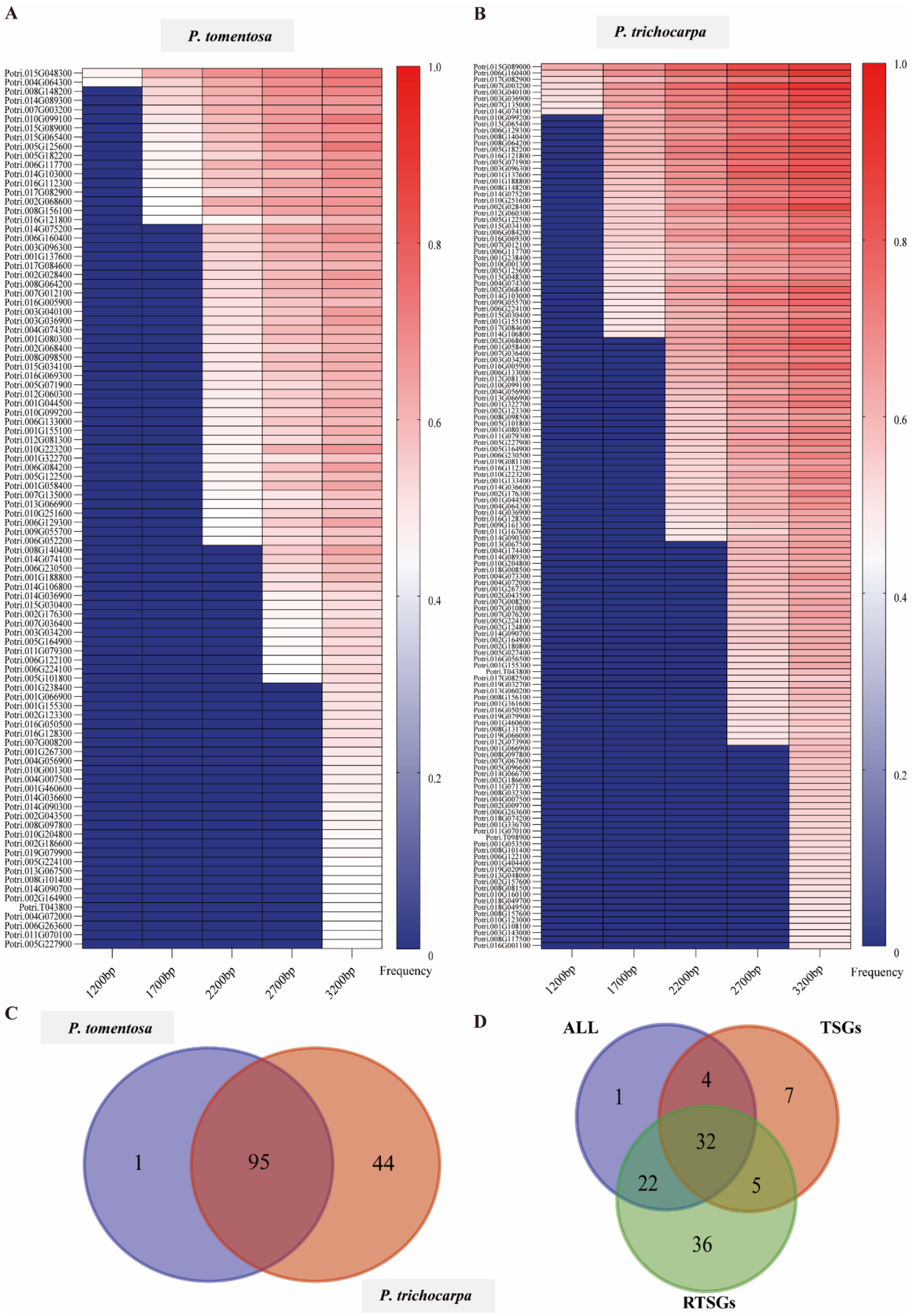

Supplementary Figure S5. Identification of conserved TFBSs in promoters of RTSGs.

(A and B) Nonredundant conserved motifs that were scanned in over 50% RFTSGs are identified in each different length of promoters of RFTSGs. For each size of promoter, the conserved motifs found using the promoter of *P. tomentosa* and *P. trichocarpa* space

are shown (P-value for conservation is indicated by color).
(C) The overlap of nonredundant conserved motifs that were scanned in over 50% RTSGs of *P. trichocarpa* and *P. tomentosa*.
(D) The veen diagram of the most conserved TFBSs at the “all genes” (All), “tissue-specific genes” (TSGs) and “root tissue-specific genes” (RTSGs) levels. In “All” and “TSGs” levels, the most conserved TFBSs are non-redundant TFBSs were identified in both poplars. In RTSGs level, the 95 most conserved TFBSs is the overlap of Fig. S5C.

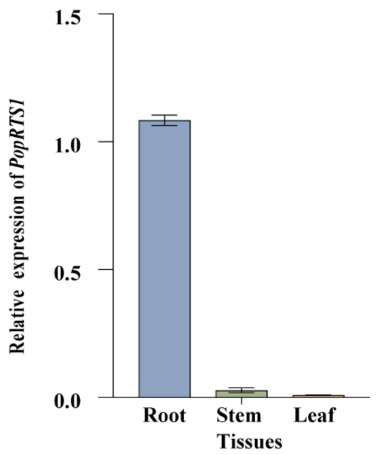

Supplementary Figure S6. Expression analysis of *PopRTS1* by qRT-PCR.
The expression analysis of *PopRTS1* in different tissues of 84k by qRT-PCR. The poplar ACTIN gene was used as an endogenous reference gene and its expression level was arbitrarily set to 1.0 for standardization. The means and standard deviations of the relative transcript levels in the respective tissue are shown.

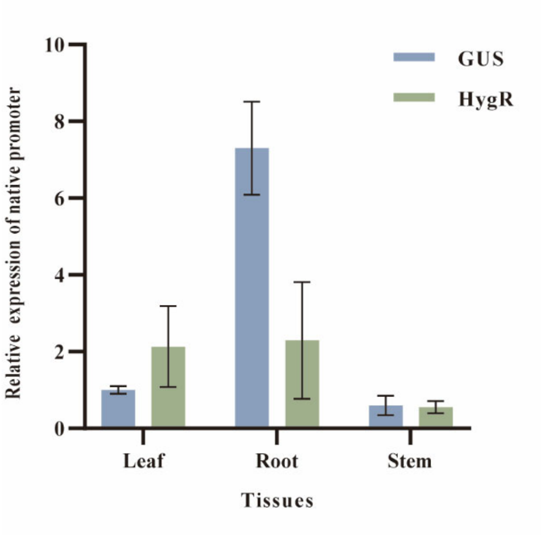

Supplementary Figure S7. Expression analysis of *GUS* driven by native promoter. The construct of native promoters fused with *GUS* for tobacco stable transformation. The poplar ACTIN gene was utilized as an endogenous reference gene and its expression level was arbitrarily set to 1.0 for standardization. The means and standard deviations of the relative transcript levels in each tissue are displayed.
