# Supplementary material for: Design and Testing of Root-Specific Synthetic Promoters by Machine Learning
Source: Int J Mol Sci. 2026 Mar 10;27(6):2540. doi: 10.3390/ijms27062540 (PMC13026494; doi:10.3390/ijms27062540)
Supplement: Supplementary file 1 [file ijms-27-02540-s001.zip › Supplementary Method S1 The processing of WGCNA data.pdf]

```r
rm(list = ls())
Expr1 <- read.table("tissuespecific.txt", header = TRUE)
Expr2 <- log(Expr1+1, 2)
dim(Expr2)

library(WGCNA) # version 1.63
library(flashClust)
library(reshape2)
library(stringr)
library(BiocParallel)
options(stringsAsFactors = FALSE)
enableWGCNAThreads()# mutiple Threads
register(MulticoreParam(60))
allowWGCNAThreads(nThreads = 60)

corType = "pearson" # correlation method,another correlation type is
"bicor"
maxPOutliers = 0.05
robustY = FALSE  # Dealing with binary data
Expr_t <- as.data.frame(t(Expr2))


sampleTree <- hclust(dist(Expr_t), method = "average")
par(mar = c(0,4,2,0))
pdf(file = "sampleTree.pdf", width = 12, height = 9)
plot(sampleTree, main = "Sample clustering to detect outliers",
sub="", xlab="")
dev.off()
# call outlier samples
clust <- cutreeStatic(sampleTree, cutHeight = 260, minSize = 8)
rownames(Expr_t)[clust==0]
keepSamples <- (clust != 0)
Expr3 <- Expr_t[keepSamples, ]
dim(Expr3)
geneNames <- colnames(Expr3)

powers <- c(seq(1, 10, by=1), seq(12, 30, by=2))
type ="unsigned"
sft = pickSoftThreshold(Expr3, powerVector=powers, networkType=type,
verbose=5)
sizeGrWindow (9,5)
par(mfrow= c(1,2))
cex1=0.9
pdf("wgcna_soft.thresholding.pdf")
```

```
plot(sft$fitIndices[,1], -
sign(sft$fitIndices[,3])*sft$fitIndices[,2],
     xlab="Soft Threshold (power)",ylab="Scale Free Topology Model
Fit,signed R^2",type="n",
     main = paste("Scale independence"))
text(sft$fitIndices[,1], -
sign(sft$fitIndices[,3])*sft$fitIndices[,2],
     labels=powers,cex=cex1,col="red")
abline(h=0.9,col="red")
dev.off()
power = sft$powerEstimate
power


net <- blockwiseModules(
  Expr3,
  maxBlockSize = dim(Expr3)[2],
  corType = corType,
  power = sft$powerEstimate,
  networkType = type,
  TOMType = type,
  saveTOMs = TRUE,
  saveTOMFileBase = "blockwiseTOM",
  minModuleSize = 30,
  mergeCutHeight =0.25,
  numericLabels = F, # modudule named in number
  nThreads = 0,
  verbose = 3)

table(net$colors)
moduleLabels = net$colors
moduleColors = labels2colors(moduleLabels)
sizeGrWindow(12,9)
par(cex = 0.6)
par(mar = c(0,4,2,0))
pdf("plotDendroAndColors222.pdf")
plotDendroAndColors(net$dendrograms[[1]],moduleColors[net$blockGenes[
[1]]],"Module colors",dendroLabels = FALSE, hang = 0.03,addGuide =
TRUE, guideHang = 0.05)
dev.off()
save(Expr3, sft, net, moduleColors, file = "wgcna-network.Rdata")

module_colors <- setdiff(unique(moduleColors), "grey")
```

```
for (color in module_colors){module <-
geneNames[which(moduleColors==color)]
  write.table(module, paste("module_",color, ".txt",sep=""),
sep="\t", row.names=FALSE, col.names=FALSE,quote=FALSE) }
# Export the network into edge and node list files Cytoscape can read
load(net$TOMFiles[1], verbose=T)
TOM <- as.matrix(TOM)
dimnames(TOM) <- list(geneNames, geneNames)


for(i in module_colors)
{
  modules = i
  probes = colnames(Expr3)
  inModule = is.finite(match(moduleColors, modules))
  modProbes = probes[inModule]
  modTOM = TOM[inModule, inModule]
  dimnames(modTOM) = list(modProbes, modProbes)
  cyt = exportNetworkToCytoscape(modTOM,edgeFile =
paste("cyt_edges_", paste(modules, collapse="-"), ".txt",
sep=""),nodeFile=paste("cyt_nodes_", paste(modules, collapse="-"),
".txt", sep=""),weighted = TRUE,threshold = 0.1, nodeNames =
modProbes, nodeAttr = moduleColors[inModule])
}
```
